# Pinpointing P450s Associated with Pyrethroid Metabolism in the Dengue Vector, *Aedes aegypti*: Developing New Tools to Combat Insecticide Resistance

**DOI:** 10.1371/journal.pntd.0001595

**Published:** 2012-03-27

**Authors:** Bradley J. Stevenson, Patricia Pignatelli, Dimitra Nikou, Mark J. I. Paine

**Affiliations:** Liverpool School of Tropical Medicine, Liverpool, United Kingdom; Centers for Disease Control and Prevention, United States of America

## Abstract

**Background:**

Pyrethroids are increasingly used to block the transmission of diseases spread by *Aedes aegypti* such as dengue and yellow fever. However, insecticide resistance poses a serious threat, thus there is an urgent need to identify the genes and proteins associated with pyrethroid resistance in order to produce effective counter measures. In *Ae. aegypti*, overexpression of P450s such as the CYP9J32 gene have been linked with pyrethroid resistance. Our aim was to confirm the role of CYP9J32 and other P450s in insecticide metabolism in order to identify potential diagnostic resistance markers.

**Methodology/Principal Findings:**

We have expressed CYP9J32 in *Escherichia coli* and show that the enzyme can metabolize the pyrethroids permethrin and deltamethrin. In addition, three other *Ae. aegypti* P450s (CYP9J24, CYP9J26, CYP9J28) were found capable of pyrethroid metabolism, albeit with lower activity. Both *Ae. aegypti* and *Anopheles gambiae* P450s (CYP's 6M2, 6Z2, 6P3) were screened against fluorogenic and luminescent substrates to identify potential diagnostic probes for P450 activity. Luciferin-PPXE was preferentially metabolised by the three major pyrethroid metabolisers (CYP9J32, CYP6M2 and CYP6P3), identifying a potential diagnostic substrate for these P450s.

**Conclusions/Significance:**

P450s have been identified with the potential to confer pyrethroid resistance in *Ae.aegypti*. It is recommended that over expression of these enzymes should be monitored as indicators of resistance where pyrethroids are used.

## Author Summary

Dengue fever, transmitted by Aedes mosquito vectors, is a major public health problem in over 100 countries. Prevention of the disease relies heavily on the use of insecticides such as pyrethroids. Unfortunately, mosquitoes are becoming resistant to these insecticides so it is urgent to identify the genes involved to develop an effective monitoring tool. In insects, P450s are critically involved in the metabolism and detoxification of insecticides. Several P450s have been found over expressed in pyrethroid resistant *Ae. aegypti*, although their functions are unknown. Here, we have expressed these enzymes in bacteria and examined their ability to metabolise pyrethroids and other compounds. From this we have identified a set of four P450s (CYPs 9J24, 9J26, 9J28 and 9J32) that are involved in pyrethroid metabolism, thus they may be considered to be key markers of resistance. To try and track their expression in field populations, we have developed diagnostic assays to monitor their activity, which will aid the development of new tools to monitor resistance and aid disease control.

## Introduction

Dengue fever, transmitted by *Aedes* mosquito vectors, is a major public health problem in over 100 countries with some 2.5 billion people at risk of the disease [1]. Prevention of the disease depends in large part on vector control, which relies heavily on the use of insecticides. Interventions are targeted mainly at the larval stage (larvicides), water source reduction, or spraying with widespread use of organophosphates such as temephos and pyrethroids such as deltamethrin. Epidemiologically, however, the adult is the most important life stage, thus insecticide treated materials (ITMs) that target this stage have been tested and show promise in reducing household level dengue vector infestations [2], [3], [4]. However, since pyrethroids are the only insecticides approved for use in ITMs [5], resistance to this insecticide class poses a serious threat to disease control; indeed, field-based resistance to pyrethroids is rapidly spreading across most parts of the world [6], [7], [8], [9]. Understanding resistance mechanisms is essential for the development of new tools to sustain vector control interventions.

Insecticide resistance in disease vectors is generally attributed to increased rates of insecticide detoxification or mutations in the target sites [10]. Due to the large numbers of potential detoxification genes in the mosquito genome (*Aedes aegypti* contains160 P450 genes [11]), detection of metabolism-based insecticide resistance is more complex than screening for specific mutations known to cause target site resistance. Nevertheless, advances in microarray and genome technology have facilitated transcriptional and genetic comparisons of susceptible and resistant populations. This has helped identify a number of P450s that are transcriptionally over-expressed in pyrethroid resistant populations of *Ae. aegypti* [11], [12], [13].

CYP9J32 in particular is a P450 that is found to be strongly linked to metabolic resistance as it is significantly over-expressed in pyrethroid resistant strains in widely separated populations in Vietnam [14], Mexico, Thailand [11] and Brazil (Strode, unpublished). Microarray screens being done in Liverpool (Barriami *et al* submitted) are continuing to indicate there may be elevated levels of several other P450s in pyrethroid resistant *Ae.aegypti* populations including CYP's 6CB1, 9J10, 9J19, 9J22, 9J24, 9J26 and 9J28. The key question is whether these P450s are capable of metabolising pyrethroids and thus functionally linked with insecticide resistance. If so they may be considered strong markers for existing or incipient metabolic resistance.

In general P450s are difficult to produce for *in vitro* analysis as they are membrane bound hemoproteins that require electrons from NADPH P450 oxidoreductase (CPR) and sometimes cytochrome b5 (b5) for catalysis [15]. However, we recently optimised the expression of mosquito P450s in *E. coli* through co-expression of *An. gambiae* CPR (AgCPR) and the addition of exogenous *A. gambiae* cytochrome b5 (Agb5) [16], [17], [18], thus facilitating their functional characterization. We have therefore adopted this approach for the expression of CYP9J32 and the other *Ae. aegypti* P450s implicated in pyrethroid resistance to investigate their ability to metabolise and thereby contribute towards insecticide resistance in *Ae. aegypti*.

## Materials and Methods

### Reagents

Oligonucleotides were synthesized by Sigma-Aldrich and enzymes for DNA manipulation were supplied by New England Biolabs. Isopropyl-ß-D-thio-galactopyranoside (IPTG), 5-aminolevulinic acid (ALA), and 3-[(3-cholamidopropyl)-dimethylammonio]-1-propanesulfonate (CHAPS) were supplied by Melford (UK). Insecticides were supplied by ChemService: 1,1,1-trichloro-2,2-di(4-chlorophenyl)ethane (DDT), (*S*)-α-cyano-3-phenoxybenzyl (1*R*,3*R*)-*cis*-2,2-dimethyl-3-(2,2-dibromovinyl)-cyclopropanecarboxylate (deltamethrin), *N*-[1-[(6-chloro-3-pyridyl)methyl]-4,5-dihydroimidazol-2-yl]nitramide (imidocloprid), 3-phenoxybenzyl (1*R,S*)-*cis*,*trans*-3-(2,2-dichlorovinyl)-2,2-dimethylcyclopropanecarboxylate (permethrin, mixture of four isomers), and 2-isopropoxyphenyl N-methylcarbamate (propoxur). (4S)-4,5-dihydro-2-(6 ′-hydroxy-2 ′-benzothiazolyl)-4-thiazolecarboxylic acid (Beetle luciferin) and proluciferin P450 substrates were supplied by Promega. HPLC solvents and ethanol were supplied by Fisher Scientific. Other chemicals were obtained from Sigma-Aldrich unless indicated otherwise.

### Gene cloning

Total RNA was extracted with either the Arcturus PicoPure Kit (Applied Biosystems) or TRI reagent (Sigma-Aldrich) from ten adult *Ae. aegypti* mosquitoes, either “Isla Mujeres” [19] or Merida strains. Complementary DNA was prepared using Superscript III (Invitrogen) with an oligo(dT)_20_ primer and used as a template for amplifying full-length genes with KOD DNA polymerase (Merk Chemicals). The gene-specific primers used in these high-fidelity PCRs were designed according to the *Ae.aegypti* genome sequence (Table 1). Amplified genes were ligated into pGEM T-easy (Promega) and sequenced. For expression, the ompA leader sequence (ompA), was engineered onto the amino-terminus to direct the P450 to the *E. coli* outer membrane during expression. The ompA-leader was inserted into the expression plasmid pCWmod1 and linked to the 5′-end of the P450 gene with codons for either: Ala-Pro by blunt ligation via an NaeI site, or Ala-Gly using cohesive ligation with the isoschizomer NgoMIV. Both linkers were tested with CYP6CB1 and no difference in expression quality or quantity was observed. All genes used cohesive ligation at the 3′-end and the restriction enzymes are indicated in Table 2. Another approach was used for CYP9J19 or CYP9J26: these genes were prepared with an ompA-Ala-Pro leader sequence by fusion PCR and cloned into pCW-ori+ using vector plasmid, pB13 [20] via NdeI and EcoRI sites as previously described [16].

**Table 1:** Accession numbers for P450 clones.

Gene	Strain	Reference*	Clone**
CYP6CB1	Isla Mujeres	XM001654530	JF924905
CYP9J10	Isla Mujeres	XM001652170	JF924906
CYP9J19	Merida	XM001652172	JF924907
CYP9J24	Isla Mujeres	XM001649048	JF924908
CYP9J26	Merida	XM001649047	JF924909
CYP9J28	Isla Mujeres	XM001649045	JF924910
CYP9J32	Isla Mujeres	XM001653404	JF924911

*: GenBank accession number for the reference genes used in primer design (Table 2).

**: GenBank accession number for the cDNA sequences isolated from the strains used for this study.

**Table 2:** Oligonucleotide sequences used for cloning *Ae. aegypti* P450 genes.

Target	Orientation	Feature	Sequence, 5′ to 3′, feature in bold
CYP6CB1	forward	ala-pro	**GCACCA**ATGTTACTTCCGATCTTACTTGTAG
CYP6CB1	reverse	EcoRI	CCTT**GAATTC**ACTTTACTTCGTCC
CYP6CB1	forward	NgoMIV	ATA**GCCGGC**ATGTTACTTCCGATCTTAC
CYP9J10	forward	NgoMIV	GTA**GCCGGC**ATGGTTGAAGTGGATTTG
CYP9J10	reverse	EcoRI	TG**GAATTC**GTTAGACATCTTTTATCAC
CYP9J24	forward	NgoMIV	GTG**GCCGGC**ATGGAGGTTAATCTGTTCTACTTC
CYP9J24	reverse	XbaI	TC**TCTAGA**CTACCCCTTTGGTCTTGGCTTG
CYP9J28	forward	NgoMIV	GGG**GCCGGC**ATGGAGGTTAATCTGTTCTATTTC
CYP9J28	reverse	EcoRI	CC**GAATTC**CTACTTCTTAGGTCTAGGTTTGAAC
CYP9J32	forward	NgoMIV	GTA**GCCGGC**ATGGAGGTGAACCTGCTTTTATTAC
CYP9J32	reverse	XbaI	CT**TCTAGA**TCACTTCCTCTTCTTAAATCTCAAATG
CYP9J19	forward	ala-pro	**GCACCG**ATGGAAGTGGATCTCCTCTCG
CYP9J19	reverse	EcoRI	**GAATTC**TACGGTATGTTAACAATCTTAAG
cyp9j19	reverse	ompA-ala-pro	GAGAGGAGATCCACTTCCAT**CGGTGCGGCCTGCGCTAC**
cyp9j26	forward	ala-pro	**GCACCA**ATGGAAGTGGAACTCCTACATGTG
cyp9j26	reverse	EcoRI site	**GAATTC**ACCGCAGCTTCAGCTCC
cyp9j26	reverse	ompA-ala-pro	CATGTAGGAGTTCCACTTCCAT**TGGTGCGGCCTGCGCTAC**

### P450 expression

Competent *E. coli* DH5α cells were co-transformed with the pCW- P450 plasmid and and pACYC-AgCPR [16]. Transformants were selected on Luria-Bertani (LB) agar plates with 50 mg/L ampicilin (pCW-P450 selection) and 34 mg/L chloramphenicol (pACYC-AgCPR selection). After overnight growth at 37°C, a single colony was used to inoculate 5 mL of LB with antibiotics (50 mg/L ampicilin and 34 mg/L chloramphenicol) for overnight growth at 37°C with shaking. Two mL of this starter culture was then used to inoculate 200 mL of Terrific Broth with antibiotics that was then incubated at 37°C with orbital shaking. Once the cultures had reached early log-phase growth (A595 = 0.8–1.0), the culture was cooled to 25°C before adding 1 mM IPTG and 0.5 mM ALA (final concentrations) and continuing incubation at 25°C with orbital shaking. Initially, cultures were monitored daily to find the incubation time for optimal P450 expression: 6CB1, 1 day; 9J19, 2 days; 9J24, 3 days; 9J26, 2 days; 9J28, 2 days; 9J32, 1 day. P450 expression was estimated by resuspending whole cells in Spectrum Buffer (100 mM Tris-HCl, pH 7.4, 10 mM CHAPS, 20% (v/v) glycerol, 1 mM EDTA) [20], adding about 1 mg/mL of sodium dithionite as a reducing agent and recording the absorption spectra (500-400 nm) change after exposing to CO for 1 min. The peak height at 450 nm was used to calculate the P450 concentration [21].

After the optimal incubation period at 25°C, the cells were harvested and membranes prepared based on the method by Pritchard *et al.* [20]. Immediately after membrane isolated by ultracentrifugation (1 hr at 180000 *g*) the sample was homogenized in TSE buffer (50 mM Tris-acetate, pH 7.6, 250 mM sucrose, 0.25 mM EDTA) using a Dounce homogenizer. Typically 1 mL of TSE was used per 200 mL of culture processed.

The membrane samples were analysed for P450 quality and content by 100-fold dilution in Spectrum Buffer and CO-difference spectroscopy [21]. Cytochrome c reductase activity was used to measure CPR content [22], and protein content was estimated by Bradford assay. Samples were stored in aliquots at −70°C.

*Anopheles gambiae* cytochrome b5 was prepared as described previously [18] .

### Insecticide metabolism

5 mM stock concentrations of insecticides were prepared in ethanol and diluted to 10× the assay concentration in 20% (v/v) ethanol immediately before each experiment to minimise precipitation of insecticide. Standard reactions contained a final ethanol concentration of 2% (v/v) with 10 µM insecticide, 0.1 µM P450, 0.8 µM cyt b5 in 200 mM Tris-HCl for pH 7.4, and NADPH regeneration components (1 mM glucose-6-phosphate (G6P), 0.25 mM MgCl_2_, 0.1 mM NADP^+^, and 1 U/mL glucose-6-phosphate dehydrogenase (G6PDH)). Reactions were started, after pre-incubation at 30°C for 5 min, by adding enzyme samples for a final reaction volume of 0.1 mL. These were incubated for a specified time at 30°C with 1200 rpm orbital shaking and quenched by adding 0.1 mL of acetonitrile. Samples were then incubated with shaking as before for an additional 10 min before centrifuging at 20000 *g* for 5 min. 0.15 mL of the supernatant was then transferred to HPLC vials, stored at room temperature and analyzed within 24 hrs. Reactions were performed in triplicate and a paired T-test of sample reactions (+NADPH) vs negative control (−NADPH) used for statistical measurements of substrate depletion.

For enzyme kinetic measurements the reaction rate in response to insecticide concentration was used to estimate Michaelis-Menton parameters. Reactions were performed, as described above, for 10 min with insecticide concentrations of: 0.25, 0.5, 1, 2, 4, 8, or 16 µM. The reactions were performed in parallel against a negative control (−NADPH) at 16 µM. Non-linear regression of results of three independent experiments were used for estimations of *K*_M_ and *k*_cat_ (SigmaPlot 11, Systat Software, Inc.).

### HPLC analysis

100 µl of acetonitrile-quenched reaction supernatant was analyzed by reverse-phase HPLC with a 250 mm C18 column (Acclaim 120, Dionex) and a mobile phase consisting of 90% acetonitrile and 10% water. The system was run at 23°C with 1 mL/min flow rate. Reactions with with permethrin, deltamethrin or DDT, were monitored by absorbance at 232 nm, whereas imidacloprid or propoxur were monitored at 270 nm. The insecticide was quantified by peak integration (Chromeleon software, Dionex). Elution times were 9.2 min for deltamethrin and 10.1 min for *cis*- and 11.9 min for *trans*-permethrin stereoisomers. There was no significant difference in the change of area for the two permethrin peaks in these reactions, therefore permethrin concentration was measured as the total area under the two peaks.

### Metabolism of probe substrates

Four resorufin ethers were tested as fluorogenic substrates and six luciferin-based substrates (P450-Glo, Promega) were tested against each of the *Ae.aegypti* P450 membrane preps. Three *An. gambiae* P450/CYP membrane preps were included for additional comparisons: CYP6Z2 [16], CYP6P3 [17], and CYP6M2 [18].

Each of these probe substrate were tested in reactions at 25°C buffered with 0.1 M KPi at pH 7.4 and included: 1 mM G6P, 0.25 mM MgCl_2_, 5 µM substrate, 0.1 or 0.2 µM P450, and cyt b5 at twice the P450 concentration. Positive reactions included 0.1 mM NADP and 1 U/mL GADPH whereas negative reactions had neither of these components and could not generate NADPH. Reactions were run in opaque white 96-well (flat-based) plates in triplicate. Luciferin (Promega) or resorufin standard curves were analyzed with each 96-well plate and used to calculate product formation rate.

Resorufin-producing reactions were monitored in a fluorescence plate-reader (Ex = 565 nm, Em = 585 nm) with 200 ms measurements recorded 24 s apart. The rate of resorufin molecules produced per P450 molecule per min (turnover) was determined by linear regression of the measurements between 2 min and 8 min after the reactions began. Luciferin reactions were run for 30 min before quenching as described by the P450-Glo kit . The endpoint signal was then measured by a luminescence plate-reader and the turnover calculated. Three replicates of positive and negative control reactions were run for each P450/substrate combination. Significant differences were determined by one-tailed T-tests (assuming equal variances).

## Results

### Expression of *Ae. aegypti* P450s in *E. coli*

**Figure 1:**
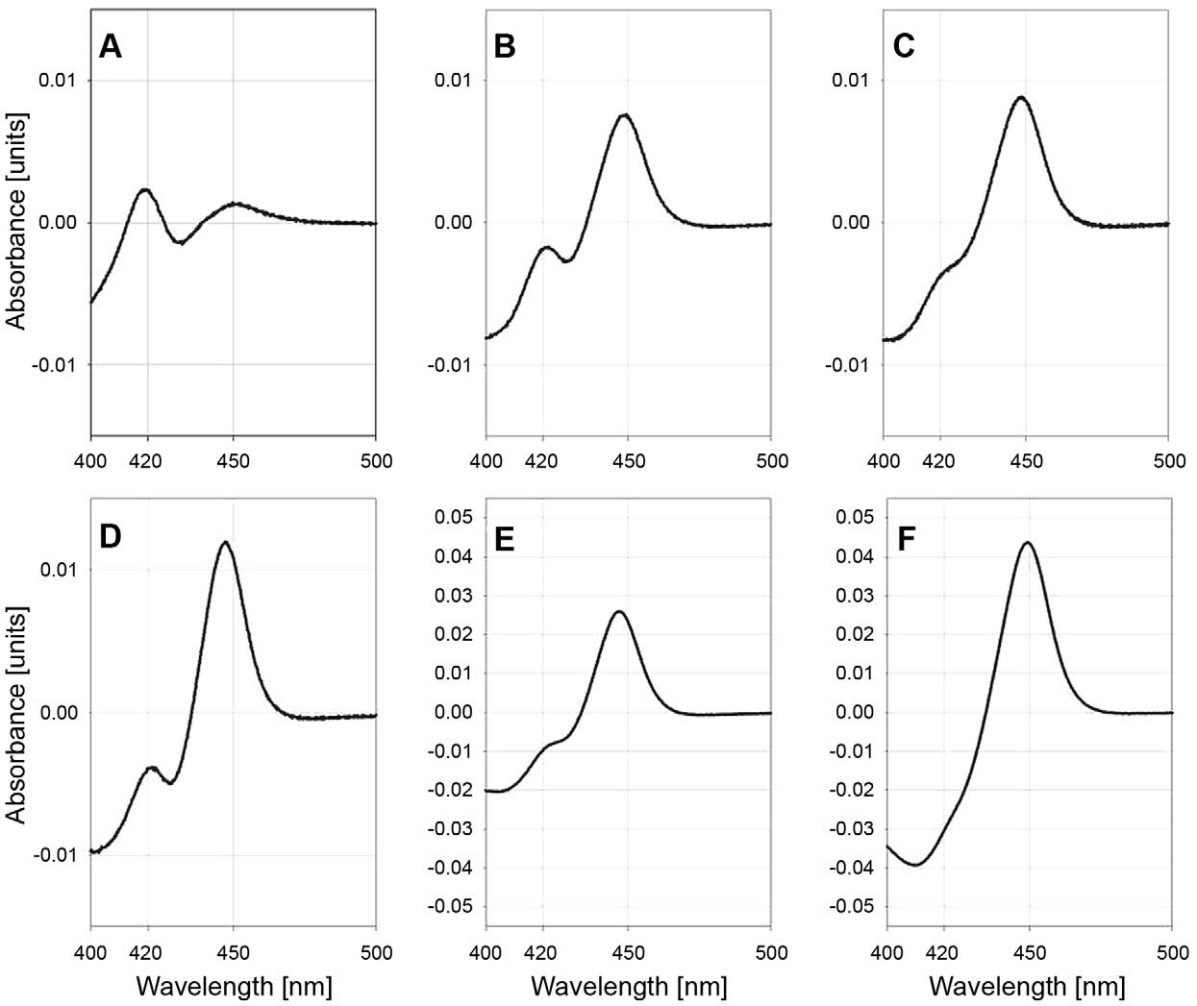
Carbon monoxide difference spectra of bacterial membranes expressing *Ae.aegypti* P450s. (A), CYP6CB1; (B), CYP9J26; (C), CYP9J32 ; (D), CYP9J24; (E), CYP9J28; and (F), CYP9J19.

To produce a catalytically active monooxygenase complex CYP's 6CB1, 9J10, 9J19, 9J24, 9J26, 9J28 and 9J32 were co-expressed with *An. gambiae* CPR in *E. coli* membranes [16]. As previously, *An. gambiae* b5 was also used to enhance P450 catalytic activity [23]; *An. gambiae* CPR and b5 are extremely similar to their *Ae. aegypti* homologues sharing 87% and 84% amino acid identity respectively, thus capable of reconstituting Aedes P450 activity. Apart from CYP9J10, which repeatedly failed to express functional enzyme in *E. coli*, all P450s produced the characteristic CO-reduced spectra indicative of active P450 (Figure 1). Apart from CYP6CB1 all P450s contained very low quantities of inactive P420 and high levels of P450 up to ∼200 nmol/L for CYP9J19 (Table 3). CYP6CB1 produced relatively low quantities of P450 (∼10 nmol/L; Table 3) and large amounts of P420, suggestive of poor enzyme quality.

**Table 3:** Yields of *Ae.aegypti* P450s expressed in *E. coli*.

P450	N^a^	Expression	Membrane Content	
		(nmol/L)^b^	nmol P450/mg^c^	nmol cyt c/min/mg^d^
CYP6CB1	3	10±7	0.1±0.1	37±24
CYP9J10	3	0	0	102
CYP9J19	1	186	1.56	32
CYP9J24	3	29±8	0.6±0.3	93±35
CYP9J26	2	25, 5	0.3, 0.1	45, 32
CYP9J28	3	47±30	1.1±0.9	157±65
CYP9J32	4	83±28	0.9±0.3	142±14

a Number of independent membranes used for yield calculations. Measurements are expressed as means ± standard deviation. Where N<3, individual measurements are listed.

b nmol of P450 isolated in membrane preparation per litre of bacterial cultures.

c P450 concentration in membrane preparations as nmol of P450 per mg of total protein.

d CPR activity in membrane preparation measured as nmol of cytochrome *c* reduced per min per mg of total protein.

### Insecticide metabolism

**Figure 2:**
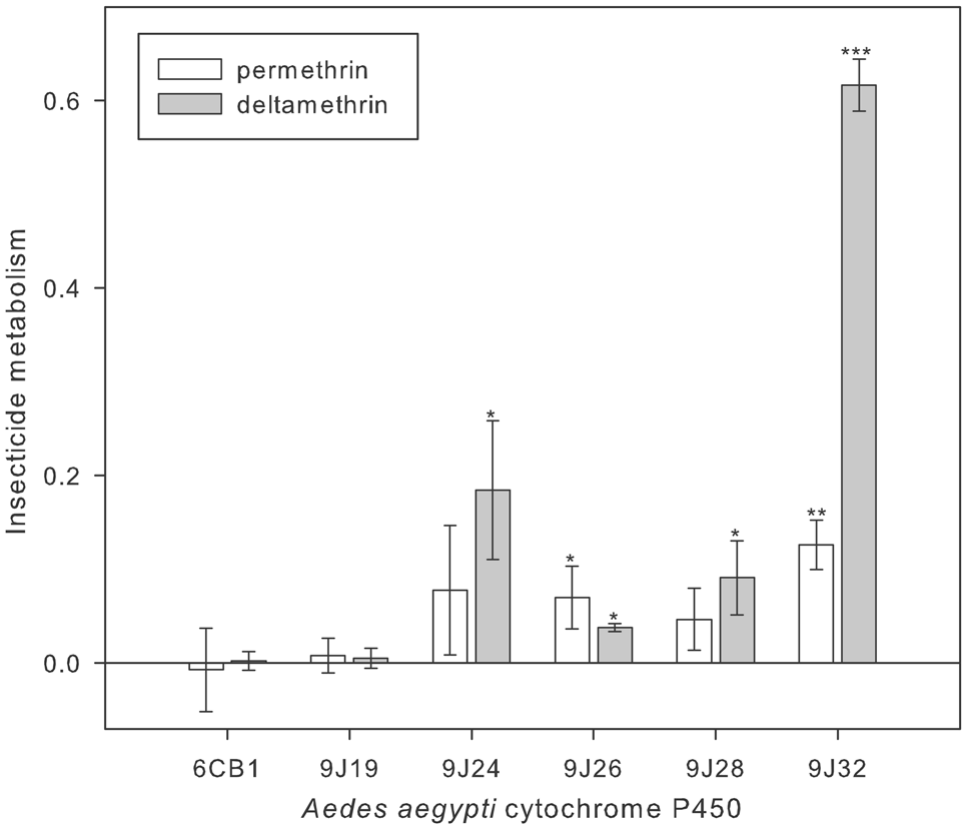
Permethrin and deltamethrin metabolism by *Ae aegypti* P450s. The proportion of 10 µM insecticide cleared by 0.1 µM P450 with 0.8 µM cyt b5 in the presence of NADPH is indicated by bar height. Error bars represent standard deviation (N = 3) and significantly greater insecticide clearance compared to negative reactions (no NADPH supplied) are indicated: *P<0.05, **P<0.01, or ***P<0.001 (paired T-test).

In order to determine if the P450s were capable of pyrethroid metabolism, they were tested against permethrin and deltamethrin, representative Type I (non-cyano) and Type II (cyano) pyrethroids respectively. Catalytic activity was assessed by measuring substrate turnover (disappearance of substrate with time)(Figure 2). CYP's 9J24, 9J26, 9J28 produced low, but reproducible deltamethrin and permethrin turnover, whereas CYP9J32 demonstrated strong activity for deltamethrin and weak activity for permethrin (Figure 2). CYP's 9J19 and 6CB1 did not metabolise permethrin or deltamethrin. The ability of CYP's 9J24, 9J26, 9J28 and 9J32 to metabolise pyrethroids was further characterised by measuring substrate dependent reaction rates. The reactions followed Michaelis-Menten kinetics and the kinetic constants were estimated for each P450-pyrethroid combination (Table 4). The apparent *K*_M_ measurements for permethrin ranged from 2.3±1.1 µM (CYP9J32) to 4.2±0.9 µM (CYP9J26) and for deltamethrin from 1.2±0.2 µM for (CYP9J26) to 5.2±2.1 µM (CYP9J32). The turnover rates (*k*_cat_) for permethrin ranged from 0.16±0.03 min^−1^ (CYP9J24) to 0.8±0.1 min^−1^ (CYP9J32) and from 0.22±0.02 min^−1^ (CYP9J26 or CYP9J28) to 3.0±0.5 min^−1^ (CYP9J32) for deltamethrin. Overall, the *K*_M_ values for all P450s were within the normal range, 1–50 µM, associated with substrate binding and P450 metabolism [18], [24]. In comparison with the normal broad range of *k*_cat_ values for P450s of 1–20 min^−1^, *k*_cat_ values were low i.e. <1 min^−1^. The exception was CYP9J32, which had a high *k*_cat_ value of ∼3 min^−1^ for deltamethrin. Interestingly the *k*_cat_ for permethrin was ∼3 fold lower (0.8 min^−1^). Finally, as for the pyrethroids, all P450s where tested against DDT (organochlorine), propoxur (carbamate), and imidacloprid (neonicotinoid), but there was no obvious substrate turnover detected.

**Table 4:** Subsrate-saturation kinetic constants.

	*K*_M_ (µM)		*K*_cat_ (min^−1^)	
P450	Permethrin	Deltamethrin	Permethrin	Deltamethrin
**CYP9J32**	2.3±1.1	5.2±2.1	0.8±0.1	3.0±0.5
**CYP9J28**	2.6±0.4	1.7±0.5	0.44±0.03	0.22±0.02
**CYP9J26**	4.2±0.9	1.2±0.2	0.6±0.1	0.22±0.01
**CYP9J24**	3.3±1.6	2.9±1.5	0.16±0.03	0.31±0.06

### Probe substrate metabolism

**Figure 3:**
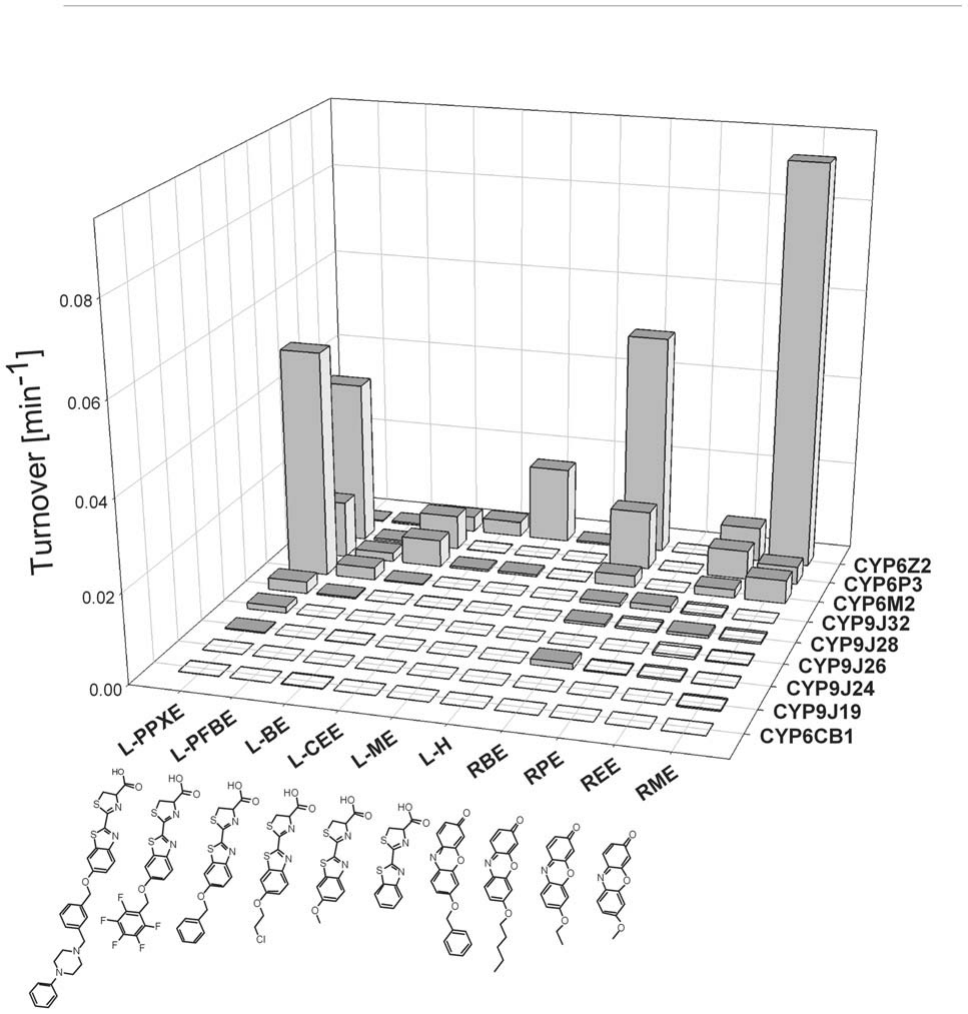
Metabolism of probe substrates by *Ae. aegypti* and *An. gambiae* P450s. Solid bars indicate significant turnover of probe substrates compared to the negative (−NADPH) control reactions (N = 3, T-test). The chemical structures of the resorufin and lucerifin-based probe substrates are shown below the X-axis.

Fluorescent and chemiluminescent substrates are routinely used in the pharmaceutical industry for monitoring P450 activity [25]. In the context of vector control these probes could be extremely useful for diagnostic monitoring of P450 levels for insecticide resistance [26]. We therefore screened the *Ae. aegypti* P450s for their ability to metabolise the four fluorogenic resorufin ethers; methyl (RME), ethyl (REE), pentyl (RPE), or benzyl ethers (RBE), as well as six P450 Glo™ proluciferin substrates, L-H, L-ME, L-CEE, L-BE, L-PFBE, L-PPXE. However, with the exception of CYP9J32, which produced a high rate of metabolism with the bulkiest luciferin compound L-PPXE, the *Aedes* P450s were largely unreactive against these substrates (Figure 3).

We extended the screen to include three *An. gambiae* P450s namely, CYP6P3 [17] and CYP6M2 [18], which metabolise pyrethroids and CYP6Z2, which does not metabolise pyrethroids [16]. Like CYP9J32, CYP6P3 and CYP6M2 showed a clear preference for the luciferin substrate L-PPXE. In contrast, CYP6Z2 showed a marked preference for the smaller probe substrates with the greatest activity towards RME for the resorufin ethers and L-ME for the proluciferins (Figure 3).

## Discussion

Over the past few years the widespread use of organophosphates and synthetic pyrethroids for the control of *Ae. aegypti* larvae and adults has fed the emergence of insecticide resistance in many dengue endemic countries [27], [28], [29]. CYP9J32 has been found over-expressed in deltamethrin and permethrin resistant *Ae aegypti* strains in Thailand, Mexico and Vietnam [11], [14]. Here we have demonstrated that CYP9J32 can metabolise both these pyrethroids. Although the production of more toxic products cannot be ruled out, P450 metabolism generally results in the production of less toxic, more excretable, hydroxylated metabolites and/or degradation products [18]. Thus, high levels of expression of CYP9J32 found in resistant populations of *Ae. aegypti* [11], [14] are indicative of a chemoprotective role for CYP9J32.

In the context of dengue control operations, CYP9J32 therefore represents a strong candidate for predicting metabolic resistance in *Ae. aegypti*, particularly in Vietnam where CYP9J32 was the only P450 significantly over-expressed in the highly deltamethrin resistant Nha Trang strain [14]. The turnover for deltamethrin was in the turnover range 1–5 min^−1^ found for other deltamethrin metabolising P450s CYP6P3 and CYP6M2 from *An. gambiae* [18], [30]. Indeed, considering the high deltamethrin resistance of this strain, what is most striking about the biochemical data is the ∼3 fold higher activity of CYP9J32 for deltamethrin (*k*_cat_ = 3.0 min^−1^ ) relative to permethrin (*k*_cat_ = 0.8 min^−1^).

While there have been a number of studies investigating insecticide resistance in different populations of *Ae. aegypti* [8], [9], [14], [19], [28], [31], it is clear that a single metabolic gene does not confer resistance in this species, unlike target site resistance. Instead, given the multiplicity of detoxification genes and their overlapping substrate specificities, numerous combinations of detoxifying enzymes may give rise to insecticide resistance [14]. Thus it is not surprising that along with CYP9J32, our data reveals at least three other *Ae. aegypti* P450s that are capable of metabolising the pyrethroid insecticides permethrin and deltamethrin, CYP9J24, CYP9J26, CYP9J28. Most significantly, CYP9J26 and CYP9J28 P450s are found over-transcribed in deltamethrin resistant field populations in both Cayman Islands and Cuba (Bariami *et al*, submitted), emphasizing a potentially important role in pyrethroid clearance *in-vivo*. Therefore, elevated levels of these P450s are a strong indicator for resistance to pyrethroids, and an important consideration for planning successful interventions [5].

At present, the identification of metabolism – based insecticide resistance generally relies on the detection of gene over-expression, which is more subjective and less accurate than identifying specific target site mutations such as kdr, which can be done by PCR [32]. Thus biochemical assays for detecting metabolic resistance by P450s are in general use [33], although they usually employ generic heme peroxidase assays that are recognised by many members of the enzyme family [7], compromising sensitivity and specificity. Having produced a panel of recombinant mosquito P450s associated with pyrethroid resistance we screened them against available fluorescent resorufin compounds and luminescent luciferin-based substrates to try and identify more specific probes for resistance monitoring. The luciferin substrate L-H has been used for tracking general P450 activity in the mosquito *Culex pipiens* [26]. Thus it was surprising that L-H proved to be such a poor substrate against the panel of individual mosquito P450s (Fig. 3). Instead, the three major pyrethroid metabolisers, CYP9J32, CYP6P3 and CYP6M2, metabolised L-PPXE (Figure 3). L-PPXE contains a noticeably large aromatic group linked to the luciferin moiety, possibly more reflective of a pyrethroid-like substrate. Consistent with their low permethrin and deltamethrin activity, it is notable that the other pyrethroid metabolisers CYP's 9J24, 9J26 and 9J28 produced low levels of PPXE activity. Given the high activity of CYP9J32 for L-PPXE this suggests that this may be a very good substrate for tracking this and potentially other pyrethroid metabolising P450s. Such an assay, requiring individual or pooled mosquito homogenates, is potentially relatively high throughgput (96 well plate) and rapid (20–30 min for enzyme reaction time), although capital expense may be a limiting factor given the high cost of luminescence detectors.

Finally, although we have not yet examined the active site structure of CYP9J32, it is worth noting that several structural models of pyrethroid metabolising mosquito P450s have been produced including CYP6M2 [18] from *An. gambiae* and CYP6AA3 and CYP6P7 from *An. minimus* [34], [35], [36]. These provide an important reference point for further studies into the mechanisms of pyrethroid metabolism by mosquito P450s.

In conclusion, we have characterised several P450s associated with insecticide resistance in *Ae. aegypti* and identified four (CYP's 9J32, 9J24, 9J26 and 9J28) that are capable of metabolising deltamethrin and permethrin, two of the commonest pyrethroids used by vector control operations. Given the escalating use of microarray and PCR-based technology for resistance monitoring [5], elevated levels of expression of these P450s should be considered a warning of incipient or existing metabolic resistance.

## References

[1] WHO (2009). Dengue and dengue hemorrhagic fever..

[2] Kroeger A, Lenhart A, Ochoa M, Villegas E, Levy M (2006). Effective control of dengue vectors with curtains and water container covers treated with insecticide in Mexico and Venezuela: cluster randomised trials.. Bmj.

[3] Lenhart A, Orelus N, Maskill R, Alexander N, Streit T (2008). Insecticide-treated bednets to control dengue vectors: preliminary evidence from a controlled trial in Haiti.. Trop Med Int Health.

[4] Vanlerberghe V, Villegas E, Oviedo M, Baly A, Lenhart A (2011). Evaluation of the effectiveness of insecticide treated materials for household level dengue vector control.. PLoS Negl Trop Dis.

[5] Hemingway J, Beaty BJ, Rowland M, Scott TW, Sharp BL (2006). The Innovative Vector Control Consortium: improved control of mosquito-borne diseases.. Trends Parasitol.

[6] Chuaycharoensuk T, Juntarajumnong W, Boonyuan W, Bangs MJ, Akratanakul P (2011). Frequency of pyrethroid resistance in Aedes aegypti and Aedes albopictus (Diptera: Culicidae) in Thailand.. J Vector Ecol.

[7] Harris AF, Rajatileka S, Ranson H (2010). Pyrethroid resistance in Aedes aegypti from Grand Cayman.. Am J Trop Med Hyg.

[8] Ahmad I, Astari S, Tan M (2007). Resistance of Aedes aegypti (Diptera: Culicidae) in 2006 to pyrethroid insecticides in Indonesia and its association with oxidase and esterase levels.. Pak J Biol Sci.

[9] Rodriguez MM, Bisset JA, De Armas Y, Ramos F (2005). Pyrethroid insecticide-resistant strain of Aedes aegypti from Cuba induced by deltamethrin selection.. J Am Mosq Control Assoc.

[10] Hemingway J, Ranson H (2000). Insecticide resistance in insect vectors of human disease.. Annu Rev Entomol.

[11] Strode C, Wondji CS, David JP, Hawkes NJ, Lumjuan N (2008). Genomic analysis of detoxification genes in the mosquito Aedes aegypti.. Insect Biochem Mol Biol.

[12] Marcombe S, Poupardin R, Darriet F, Reynaud S, Bonnet J (2009). Exploring the molecular basis of insecticide resistance in the dengue vector Aedes aegypti: a case study in Martinique Island (French West Indies).. BMC Genomics.

[13] Poupardin R, Reynaud S, Strode C, Ranson H, Vontas J (2008). Cross-induction of detoxification genes by environmental xenobiotics and insecticides in the mosquito Aedes aegypti: impact on larval tolerance to chemical insecticides.. Insect Biochem Mol Biol.

[14] Bingham G, Strode C, Tran L, Khoa PT, Jamet HP (2011). Can piperonyl butoxide enhance the efficacy of pyrethroids against pyrethroid-resistant Aedes aegypti?. Trop Med Int Health.

[15] Paine MJI, Scrutton NS, Munro AW, Roberts GCK, Wolf CR, Ortiz de Montellano PR (2004). Electron Transfer Partners of Cytochrome P450.. Cytochromes P450: Stucture, Mechanism and Biochemistry. 3rd ed.

[16] McLaughlin LA, David J-P, Vontas J, Niazi U, Bibby J (2008). Characterization of *Anopheles gambiae* CYP6Z2: role in xenobiotic metabolism and insecticide resistance.. Insect Molecular Biology.

[17] Muller P, Chouaibou M, Pignatelli P, Etang J, Walker ED (2008). Pyrethroid tolerance is associated with elevated expression of antioxidants and agricultural practice in Anopheles arabiensis sampled from an area of cotton fields in Northern Cameroon.. Mol Ecol.

[18] Stevenson BJ, Bibby J, Pignatelli P, Muangnoicharoen S, O'Neill PM (2011). Cytochrome P450 6M2 from the malaria vector Anopheles gambiae metabolizes pyrethroids: Sequential metabolism of deltamethrin revealed.. Insect Biochem Mol Biol.

[19] Flores AE, Grajales JS, Salas IF, Garcia GP, Becerra MH (2006). Mechanisms of insecticide resistance in field populations of Aedes aegypti (L.) from Quintana Roo, Southern Mexico.. J Am Mosq Control Assoc.

[20] Pritchard MP, Glancey MJ, Blake JA, Gilham DE, Burchell B (1998). Functional co-expression of CYP2D6 and human NADPH-cytochrome P450 reductase in Escherichia coli.. Pharmacogenetics.

[21] Omura T, Sato R (1964). The carbon monoxide-binding pigment of liver microsomes. I. Evidence for its hemoprotein nature.. Journal of Biological Chemistry.

[22] Vermilion JL, Coon MJ (1978). Purified liver microsomal NADPH-cytochrome P-450 reductase. Spectral characterization of oxidation-reduction states.. J Biol Chem.

[23] Schenkman JB, Jansson I (1999). Interactions between cytochrome P450 and cytochrome b5.. Drug Metab Rev.

[24] McLaughlin LA, Paine MJ, Kemp CA, Marechal JD, Flanagan JU (2005). Why is quinidine an inhibitor of cytochrome P450 2D6? The role of key active-site residues in quinidine binding.. J Biol Chem.

[25] Cohen LH, Remley MJ, Raunig D, Vaz AD (2003). In vitro drug interactions of cytochrome p450: an evaluation of fluorogenic to conventional substrates.. Drug Metab Dispos.

[26] Inceoglu AB, Waite TD, Christiansen JA, McAbee RD, Kamita SG (2009). A rapid luminescent assay for measuring cytochrome P450 activity in individual larval Culex pipiens complex mosquitoes (Diptera: Culicidae).. J Med Entomol.

[27] Cui F, Raymond M, Qiao CL (2006). Insecticide resistance in vector mosquitoes in China.. Pest Manag Sci.

[28] Lima EP, Paiva MH, de Araujo AP, da Silva EV, da Silva UM (2011). Insecticide resistance in Aedes aegypti populations from Ceara, Brazil.. Parasit Vectors.

[29] Llinas GA, Seccacini E, Gardenal CN, Licastro S (2010). Current resistance status to temephos in Aedes aegypti from different regions of Argentina.. Mem Inst Oswaldo Cruz.

[30] Müller P, Warr E, Stevenson BJ, Pignatelli PM, Morgan JC (2008). Field-caught permethrin-resistant *Anopheles gambiae* overexpress CYP6P3, a P450 that metabolises pyrethroids.. PLOS Genetics.

[31] Saavedra-Rodriguez K, Urdaneta-Marquez L, Rajatileka S, Moulton M, Flores AE (2007). A mutation in the voltage-gated sodium channel gene associated with pyrethroid resistance in Latin American Aedes aegypti.. Insect Mol Biol.

[32] Bass C, Nikou D, Donnelly MJ, Williamson MS, Ranson H (2007). Detection of knockdown resistance (kdr) mutations in Anopheles gambiae: a comparison of two new high-throughput assays with existing methods.. Malar J.

[33] Hemingway J, Hawkes NJ, McCarroll L, Ranson H (2004). The molecular basis of insecticide resistance in mosquitoes.. Insect Biochem Mol Biol.

[34] Lertkiatmongkol P, Jenwitheesuk E, Rongnoparut P (2011). Homology modeling of mosquito cytochrome P450 enzymes involved in pyrethroid metabolism: insights into differences in substrate selectivity.. BMC Res Notes.

[35] Boonsuepsakul S, Luepromchai E, Rongnoparut P (2008). Characterization of Anopheles minimus CYP6AA3 expressed in a recombinant baculovirus system.. Arch Insect Biochem Physiol.

[36] Duangkaew P, Pethuan S, Kaewpa D, Boonsuepsakul S, Sarapusit S (2011). Characterization of mosquito CYP6P7 and CYP6AA3: differences in substrate preference and kinetic properties.. Arch Insect Biochem Physiol.

